# Co-Designing Age-Friendly Health System Materials With Older Adults and Caregivers

**DOI:** 10.1093/geroni/igaa057.2398

**Published:** 2020-12-16

**Authors:** Erin Emery-Tiburcio, Carol Regan, Judith Hilman, Michelle Newman

**Affiliations:** 1 Rush University Medical Center, Chicago, Illinois, United States; 2 Community Catalyst, Boston, Massachusetts, United States; 3 Policy Catalyst, Salt Lake City, Utah, United States

## Abstract

Achieving better care for older adults requires that they advocate for their own health needs. As health systems become Age-Friendly, education for older adults about the 4Ms is essential. Community Catalyst (CC) is working with older adults at six GWEPs to co-design materials explaining the interconnected 4Ms. In November 2019, CC hosted two focus groups (n=17) with older adults from Rush University Medical Center to understand their views on health care and introduce the 4M concepts. Key themes included the importance of being listened to, of each M and how they are connected, and for materials with graphics and personal stories. Based on this information, prototype educational materials were developed and three additional groups were held in February 2020 (n=30, including family caregivers) to solicit feedback on content and design. The co-design process, evaluation methods, and implications of including older adults in educational design will be discussed.

